# An fMRI Study of the Impact of Block Building and Board Games on Spatial Ability

**DOI:** 10.3389/fpsyg.2016.01278

**Published:** 2016-08-29

**Authors:** Sharlene D. Newman, Mitchell T. Hansen, Arianna Gutierrez

**Affiliations:** Department of Psychological and Brain Sciences, Indiana UniversityBloomington, IN, USA

**Keywords:** spatial processing, game play, fMRI, block building, mental rotation

## Abstract

Previous studies have found that block play, board games, and puzzles result in better spatial ability. This study focused on examining the differential impact of structured block play and board games on spatial processing. Two groups of 8-year-old children were studied. One group participated in a five session block play training paradigm and the second group had a similar training protocol but played a word/spelling board game. A mental rotation task was assessed before and after training. The mental rotation task was performed during fMRI to observe the neural changes associated with the two play protocols. Only the block play group showed effects of training for both behavioral measures and fMRI measured brain activation. Behaviorally, the block play group showed improvements in both reaction time and accuracy. Additionally, the block play group showed increased involvement of regions that have been linked to spatial working memory and spatial processing after training. The board game group showed non-significant improvements in mental rotation performance, likely related to practice effects, and no training related brain activation differences. While the current study is preliminary, it does suggest that different “spatial” play activities have differential impacts on spatial processing with structured block play but not board games showing a significant impact on mental rotation performance.

## Introduction

Play is an important way that young children learn (Singer et al., 2006). Playing with spatial toys and engaging in spatial activities may prove to be an essential part of the development of spatial thinking. There are a number of studies that have related spatial play with spatial skill (Levine et al., 2012; Jirout and Newcombe, 2015) and number processing (Cheng and Mix, 2014; Verdine et al., 2014; Casey et al., 2015). For example, in a recent study by Jirout and Newcombe (2015) a large group of 4- to 7-year-old children were studied. There it was found that those who frequently participated in block play, puzzles, and board games had higher spatial ability than those who participated more in other activities like drawing, playing with sound-producing toys, trucks, and riding bikes.

While studies seem to suggest a relationship between games like block building, board games, and puzzles to spatial processing, there are few studies that have explored the differential impact of these spatial games on spatial processing. There is some indication that they are not equivalent. One of the very few studies directly comparing spatial games examined their impact on mathematics. Cheng and Mix (2014) examined the effect of two types of spatial training (mental rotation and puzzles) in 7-year-old children. Both mental rotation and puzzle play have been suggested to impact spatial skill. They found that only mental rotation training resulted in improvements in performance on missing-term mathematics problems (e.g., 2 + __ = 7); but mental rotation training failed to improve place-value understanding. The differential effect of puzzles and mental rotation was observed on mathematics and not directly on spatial thinking. However, the results observed by Cheng and Mix demonstrate that different types of spatial training have different consequences and further that different types of spatial play may have a different impact on spatial processing.

The current study is a preliminary examination of the differential neural and behavioral impact of playing a structured block building and board game on spatial processing. The effect of a 5 day, 30 min per day training, with 8-year-old children was examined using both behavioral and functional magnetic resonance imaging (fMRI). Play was used here as it is an activity that children engage in regularly. We hypothesized that while both block play and board games may result in improvements in some aspects of spatial thinking, the impact of these two games will vary, as shown by Cheng and Mix (2014). Both games examined are commercially available games—Blocks Rock! and Scrabble.

### Block play

Block play has garnered a great deal of attention in terms of its potential link to spatial thinking (Casey et al., 2008). There are at least two key types of spatial skills closely related to block building—spatial visualization and mental rotation. Spatial visualization involves mentally combining objects to produce designs. As an individual is working with blocks, he or she is mentally visualizing how blocks will fit and interact with one another. Also the blocks are three-dimensional objects. Understanding how these complex objects fit together and relate to each other in designs that can be built either flat on the table or up off of the table provides additional spatial perspective to improve visualization. The second skill, mental rotation, involves mentally visualizing what an object will look like after it is rotated (Casey et al., 2008). Piaget and Inhelder (1971) proposed that children under seven were unable to perform mental rotation or dynamic imagery. However, more recent work has found that this proposal is incorrect. Mental rotation develops in infancy (Quinn and Liben, 2008; Moore and Johnson, 2011). Additionally it has been shown that manually interacting with objects improves a child's ability to mentally rotate it (Möhring and Frick, 2013; Frick and Wang, 2014) with infants who are mobile having better mental rotation ability. This suggests that physically interacting with the blocks during block play may also be an important aspect of the game.

Although, many preschool and elementary programs as well as homes have block toys, how these toys are played with has an impact on whether and how spatial skills are developed. Two types of block play have been considered, free play where children are provided blocks and they create designs, and structured block play in which children build a model of a structure (Verdine et al., 2014). It is structured block play that has been suggested to require the analysis of a spatial representation and that may result in more significant improvements in spatial ability. By spatial analysis we mean the ability to specify the parts and the overall configuration of an object and to understand how the parts are related to form a whole. It includes the ability to segment an object into parts and to integrate those parts into a coherent whole (e.g., Delis et al., 1986, 1988; Stiles and Stern, 2009). As mentioned above, the blocks are 3D and the structures built are 3D. During structured block play a 2D picture of a structure is copied. In other words, a 2D-3D spatial transformation is performed. Training in 2D-3D spatial transformations has been found to improve mental rotation performance in girls (Tzuriel and Egozi, 2007, 2010). This suggests another mechanism by which structured block play may improve spatial processing. Again, while classrooms may have block building activities, there is not enough structured play for children to greatly enhance spatial learning (Casey et al., 2015). Casey et al. (2015) suggests that “if this skill were taught in a more systematic way in the early childhood classroom, it might have the potential to further develop spatial reasoning.” The primary goal of the current study was to further explore the impact of structured block play on spatial ability in young elementary school children.

### Board games

In addition to block play, board games, and puzzles have also been linked to improved spatial processing (Ramani and Siegler, 2008; Siegler and Ramani, 2009; Jirout and Newcombe, 2015). For example, Siegler and Ramani have shown that number knowledge is improved in preschoolers who played a linear number-based board game like Chutes and Ladders. They also reported that the gains in number knowledge persisted. There are a number of different types of board games with some focusing on counting while others, like Scrabble, focusing on spelling. Board games that focus on spelling are an interesting category because Jirout and Newcombe (2015) found that playing word and spelling games with parents had a marginally significant effect on Block Design score. A possible explanation for such a relationship is that the spatial relationships between letters are extremely important for spelling. For example, “biek” is not a word while “bike” is because the spatial relationship between the “k” and “e” is important. Spelling games like Scrabble also use spatial language (e.g., building words up or down) which draws the child's attention to spatial relationships. Additionally, Scrabble, like structured block play provides a similar sensory-motor experience due to the hand-eye movements necessary to manipulate pieces (letters) and place them on the board. This sensory-motor experience has been found to be important to spatial processing (Ballard et al., 1992).

While there are some similarities between structured block play and board games like Scrabble, there are also differences. For example, structured block play requires the building of complex spatial configurations and more explicitly focuses on spatial analysis and spatial working memory. Scrabble, on the other hand, focuses on word creation from a jumbled set of letters. Therefore, while Scrabble has spatial components (e.g., words are spatially organized letters and the use of spatial language), it does not require spatial working memory processes related to holding a non-verbal spatial configuration in short-term memory. This difference predicts that different brain regions will be recruited during structured block play and Scrabble. One such region is the parahippocampus which has been linked to spatial memory (Johnsrude et al., 1999; Bohbot et al., 2000; Burgess et al., 2001). By recruiting spatial processing regions like the parahippocampus during structured block play these regions begin to develop their processing strategy which may result in differential involvement during mental rotation before and after structured block play training.

### Current study

Based on previous research the hypothesis tested here is that structured block play will result in greater spatial processing gains than board games and will therefore have different behavioral and neural consequences. Greater gains in spatial processing for block play are thought to be due to its emphasis on spatial analysis; this includes determining the spatial relationships between parts and its emphasis on spatial working memory. To test this hypothesis we employed a combination of behavioral and neuroimaging methods. A 2D letter mental rotation task was used to test spatial processing. Mental rotation is a test of spatial visualization and analysis which has long been used as a measure of spatial processing ability. Because of the age of our population (8-years) the complexity (2D) and familiarity (letters) of the objects to be rotated were simplified (Kosslyn et al., 1990; Lütke and Lange-Küttner, 2015). By simplifying the stimuli the objects to be rotated were easier to encode and identify (Bialystok, 1989). It was also important to use stimuli different from the blocks used during block play. There has been some debate regarding whether spatial training transfers to other tasks and stimuli (Kail, 1986; Kail and Park, 1990). Scrabble may be expected to have an advantage during mental rotation here due to the use of letter stimuli; therefore a greater increase in performance by the block play group will demonstrate that the training not only transfers to another task but also to other object stimuli.

## Methods

### Participants

Thirty-six (male = 21) 8-year-old children participated in this study (see Table 1 for demographic information). All were typically developing children with no history of neurological disorders. Six participants were excluded due to excessive motion in the scanner (>5 mm) and three were lost to attrition, leaving 28 (male = 15) total usable imaging participants. Of the 28, 14 (male = 8) were placed in the block play group and 14 (male-7) were placed in the board game group. Parents completed a short survey regarding their child's play behavior and parental education level (see Appendix). Participants had a variety of block building experiences (e.g., playing with Legos) prior to this study (information was obtained from parental survey); as such the two groups were equated on spatial play. Therefore, the groups were balanced on gender, age, mathematics test score, parental education and the amount of previous spatial play. Parental consent and child assent were both obtained prior to the first experimental session, in accordance with the Indiana University Institutional Review Board.

**Table 1:** **Demographic information**.

	**Block play**	**Board game**	***t*-value**
Age (years)	8 years; 3 months ± 0.29	8 years; 2 months ± 0.17	<1
% Female	46%	43%	
Math test	53% ± 25%	47% ± 24%	<1
Parent education	Bachelor's degree	Bachelor's degree	

Age, the age of the children; % Female, the proportion of subjects in each group who were girls; Math test, score on an abbreviated test of mathematics ability; Parent education, average level of education of the participant's parents ranging from high school to graduate/professional school.

### Experimental design

The participants took part in seven sessions, all on separate days. The first and last sessions were pre- and post-training evaluations. The middle five sessions were the training sessions. The mean number of days between the first and final session was similar for both groups (blocks play: 12.7 ± 4.4; Scrabble: 12 ± 6.5; *p* = 0.36). MRI scanning was performed during the pre- and post-training sessions. All sessions took place in the Department of Psychological and Brain Sciences at Indiana University.

### Pre-training

Parents completed a survey to obtain information regarding the child's prior play activities, musical training, and number/mathematics activities as well as demographic information and history of learning disorders (see Appendix for survey details). The groups were constructed in an attempt to equate them on these measures. Musical training, mathematics skill, gender as well as socio-economic status, for which parental education is a proxy, have all been linked to spatial processing. Mathematics competency was assessed by using a subset of questions from the Grade 2 Mathematics California Standards Tests from 2003 to 2007. Questions from the Number Sense and Algebra and Functions sections were used. Participants were given 15 min to complete as many of the 24 questions as possible. Parental education was averaged within each group.

**Figure 1:**
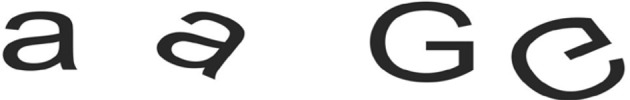
**Example stimuli**. Both upper and lower case letters were presented. The difficulty was manipulated by varying the angle of rotation (left = easy, low degree of rotation and right = hard, large angle of rotation).

The scanner task was a mental rotation task. A pair of letters was presented. The letter on the left was oriented in a normal upright position, and the letter on the right was either rotated and non-mirrored or rotated and mirrored. The angle of rotation varied from 30 to 180° (see Figure 1). Easy problems had an angle of rotation that was 90° or less. If the right letter was only rotated, the letter pair was considered the *same* and the participant pressed a button with their right index finger. If the right letter was rotated and mirrored then the pair was considered *different* and they pressed a button with their left index finger. Half of the trials were same and half were different. A block design was employed. Each block contained six letter pairs, and the blocks were separated by 12 s fixation periods. The blocks contained a mixture of *same* and *different* pairs and angles of rotation. If the participant did not answer a trial within 10 s, the program moved to the next trial. There were 24 s fixation periods to start and end the mental rotation task. The duration of the mental rotation task was 360 s. Accuracy and reaction time were recorded.

The pre-training mental rotation performance was also used to match groups. A more lenient one-tailed *t*-test was used to compare performance across groups. Prior to training the groups showed no differences in RT for easy (*p* = 0.2), hard (*p* = 0.28), or when they were combined (*p* = 0.47) on the mental rotation task. There were also no differences in accuracy for easy (*p* = 0.37), hard (*p* = 0.11), or when they were combined (*p* = 0.14).

### Training

Participants were separated into one of two groups—block play with the game Blocks Rock! and a board game, Scrabble. Both games are commercially available and both games are competitive games in which two players interact. The Blocks Rock! game has a set of cards, two identical sets of blocks of varying shape, size, and color and a bell. Each player has a set of blocks and one player turns over a card during play that has a particular structure, point value, and how to build the structure (e.g., up or flat on the table). The complexity of the structure increases during play. Each player attempts to build the structure as fast as possible with the player who does so correctly first and rings the bell being awarded the points displayed on the card. The score is kept and once all cards have been played the winner is the player with the most points. Scrabble is a popular competitive word game and the standard rules were used during play. During each training session, participants played either Blocks Rock! or Scrabble for 30 min with either another participant matched for skill level or a research assistant who adjusted their play to match the subject. Score was kept for each training session for motivational purposes.

### Post-training

Participants played the same game from their training sessions for 15 min before completing the MRI portion of the session. The MRI protocol for the post-training session was identical to that of the pre-training session.

### Imaging parameters

Participants underwent MRI scanning using a 12-channel head coil and a Siemens 3T Tim Trio MRI scanner. The first scan was an anatomical T1-weighted scan used to co-register functional images. An MPRAGE sequence (192 sagittal slices; FOV = 256 mm, matrix = 256 × 256, TR = 1800 ms, TE = 2.67 ms, TI = 900 ms, flip angle = 9°, slice thickness = 1 mm, resulting in 1 × 1 × 1-mm voxels) was used. The experimental functional scan was a multiband EPI scan (33 axial slices using the following protocol: field of view = 192 mm, matrix = 128 × 128, iPAT factor = 2, TR = 2000 ms, TE = 30 ms, flip angle = 60°, slice thickness = 3.8 mm, 0 gap).

### Data analysis

fMRI data were analyzed with SPM8 (Wellcome Trust Centre for Neuroimaging; http://www.fil.ion.ucl.ac.uk/spm). fMRI data were preprocessed in several steps including slice timing correction, motion correction by realignment, co-registration between functional and anatomical scans, spatial normalization, and smoothing. All functional data were resampled to 2 mm^3^ isomorphic voxels normalized to the Montreal Neurological Institute (MNI) template. For spatial smoothing an 8 mm FWHM Gaussian kernel was applied. On the preprocessed fMRI data of individual subjects, a canonical statistical analysis based on the general linear model (GLM) and Gaussian random field theory was performed (Friston et al., 1995). The hemodynamic response for the stimuli blocks were modeled with a canonical HRF built on the onsets of the blocks with the block duration included in the analysis. For each individual data analysis, regressors were built for the mental rotation blocks, fixation blocks, and six regressors from the realignment step were included in the model to remove unexpected effects from noise from head movement.

In order to examine the activation related to mental rotation at each time point and for each group contrast images for mental rotation compared to fixation were computed. This was performed to allow for inspection of the results prior to group comparisons to ensure analysis quality. Next the effect of training was examined for each group separately. The mental rotation minus fixation contrasts for each timepoint were analyzed using a paired *t*-test to compare the pre- and post-training activation for each group. Additionally, to examine activation differences between groups at each timepoint one-sample *t*-tests were performed (e.g., block play groups minus board game group for the post-training scan).

For the contrasts examined we applied a Monte Carlo simulation of the brain volume to establish an appropriate voxel contiguity threshold. The threshold obtained from the simulation has the advantage of higher sensitivity to smaller effect sizes (Slotnick and Schacter, 2004). The result of the Monte Carlo simulation indicated that a cluster size of 20 contiguous resampled voxels using an uncorrected threshold of *p* < 0.005 would be appropriate to control type I error, at *p* < 0.05 corrected for the multiple comparisons in the whole brain volume analysis.

## Results

### Behavioral (reaction time)

**Figure 2:**
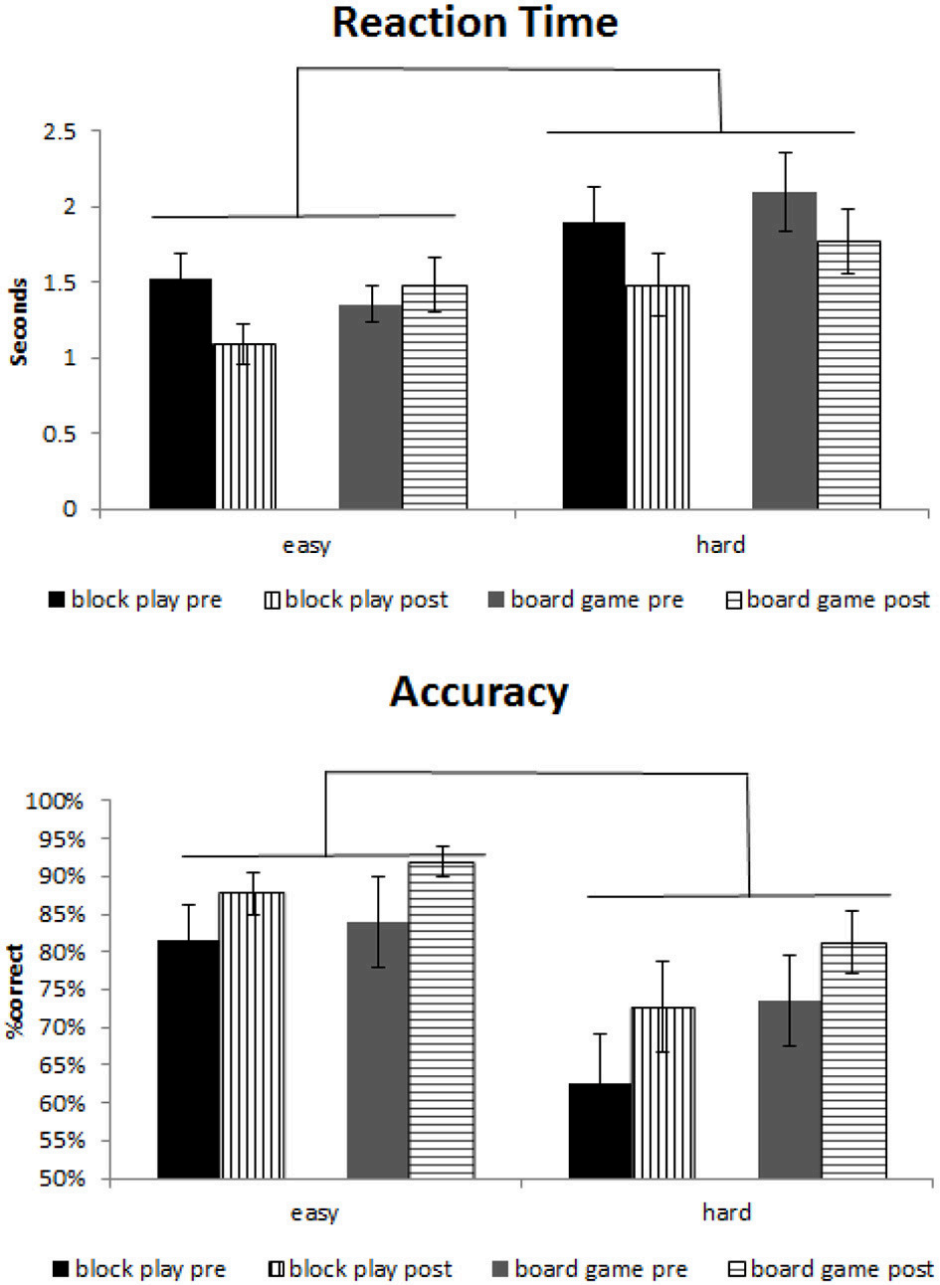
**Mental rotation behavioral results**. There is a main effect of difficulty and training. While both groups showed some improvements in performance post training, only the block play group showed significant RT and accuracy improvements after training. Error bars depict standard error.

A 2 (block play vs. board game) × 2 (pre vs. post) × 2 (easy vs. difficult) between subjects ANOVA was performed on the RT data using SAS 9.4. The results showed a significant effect of training [*F*_(1, 108)_ = 3.78; *p* = 0.054; η^2^ = 0.034]; and difficulty [*F*_(1, 108)_ = 10.5; *p* = 0.002; η^2^ = 0.089]. Although none of the interactions were found to be significant, because our a priori hypothesis was that the block play group would show an effect of training but not the board game group and because there was a trend of an interaction between group and training for RT (see Figure 2), we examined each group separately using a within-subjects ANOVA. The block play group showed a significant effect of training [*F*_(1, 14)_ = 8.92; *p* = 0.0098; η^2^ = 0.083] and a significant effect for difficulty [*F*_(1, 14)_ = 771.76; *p* = 0.023; η^2^ = 0.067]. The interaction between training and difficulty was not significant. The board game group failed to show an effect of training [*F* < 1]. An effect of difficulty was also not observed [*F*_(1, 13)_ = 5.12; *p* = 0.26; η^2^ = 0.11]. Also, the board game group showed an interaction between training and difficulty [*F*_(1, 13)_ = 5.75; *p* = 0.032; η^2^ = 0.024]. See Supplemental Data for complete behavioral statistics.

### Behavioral (accuracy)

The same 2 (block play vs. board game) × 2 (pre vs. post) × 2 (easy vs. difficult) between subjects ANOVA was performed on the accuracy data. The results showed a significant effect of training [*F*_(1, 108)_ = 5.06; *p* = 0.027; η^2^ = 0.045], difficulty [*F*_(1, 108)_ = 15; *p* = 0.0002; η^2^ = 0.12]; and a trending effect of group [*F*_(1, 108)_ = 3.36; *p* = 0.069; η^2^ = 0.03]. Although none of the interactions were found to be significant, because our a priori hypothesis was that the block play group would show an effect of training but not the board game, we examined each group separately using a within-subjects ANOVA. The block play group showed a significant effect of training [*F*_(1, 14)_ = 5.75; *p* = 0.031; η^2^ = 0.0422] and a trending effect of difficulty for accuracy [*F*_(1, 14)_ = 76.71; *p* = 0.072; η^2^ = 0.16]. The interaction between training and difficulty was not significant. The board game group failed to show an effect of training [*F*_(1, 13)_ = 3.51; *p* = 0.084; η^2^ = 0.048] while the effect was trending. An effect of difficulty was observed for accuracy [*F*_(1, 13)_ = 89,401; *p* = 0.0021; η^2^ = 0.085].

### fMRI

**Figure 3:**
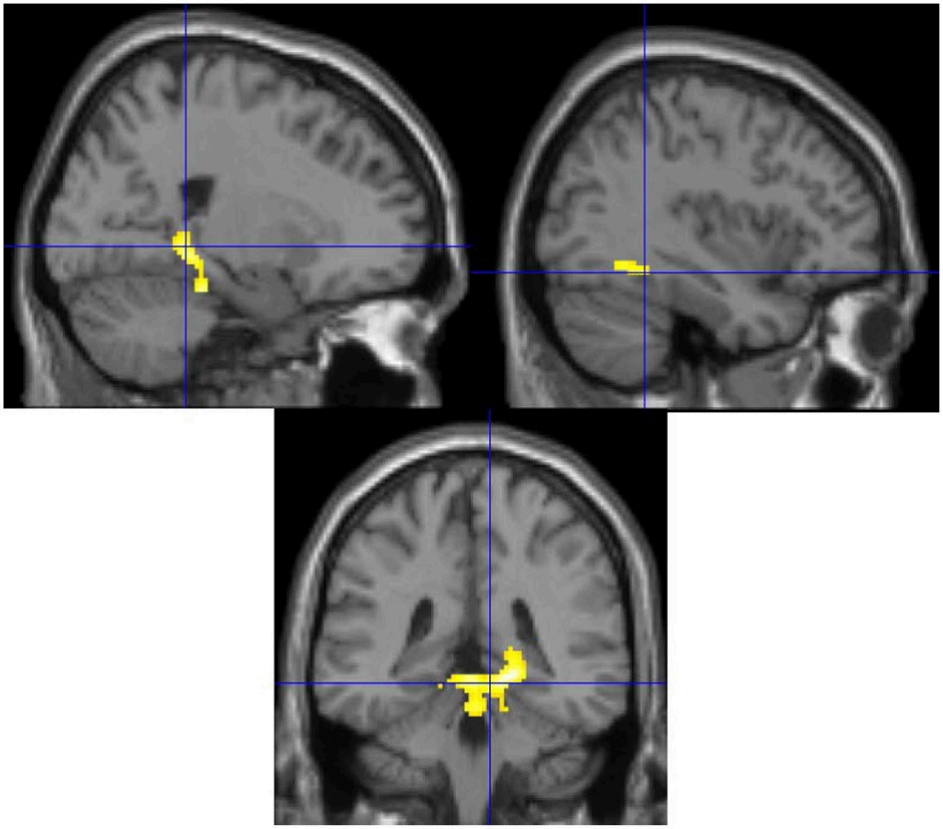
**The post- minus pre-training contrast for the block play group**. Increased activation is observed in the anterior lobe of the cerebellum, the parahippocampus and the fusiform gyrus after training for the block play group.

To examine the effect of training in each group, the post-training minus pre-training contrast was examined. It showed that the block play group had increased activation in the anterior lobe of the cerebellum extending into the right parahippocampus and the bilateral fusiform gyrus (see Figure 3; Table 2) after block play training than prior to training. The board game group failed to show any significant activation when comparing the pre- and post-scans, analogous to the behavioral finding of no effects of training.

**Table 2:** **Activation details for significant activation clusters**.

**Region**	**BA**	***k***	***t***	**MNI coordinates**		
				***x***	***y***	***z***
**BLOCK PLAY (POST- MINUS PRE-TRAINING)**						
Right cerebellum anterior lobe		590	4.92	16	−32	−10
Right cerebellum anterior lobe			4.81	20	−30	−20
Left cerebellum anterior lobe			4.26	0	−38	−8
Left fusiform gyrus	37	34	3.53	−36	−54	−10
Left fusiform gyrus	37		3.4	−36	−46	−12
Right fusiform gyrus	37	20	3.36	36	−42	−8
**POST-TRAINING (BLOCK PLAY GROUP MINUS BOARD GAME GROUP)**						
Left medial frontal gyrus	9	37	3.73	−8	50	32
Left precentral gyrus	6	13	3.14	−44	−6	24

BA, Broadman's area; k, cluster extent; t, t-test; x, y, z are coordinates in standardized space.

**Figure 4:**
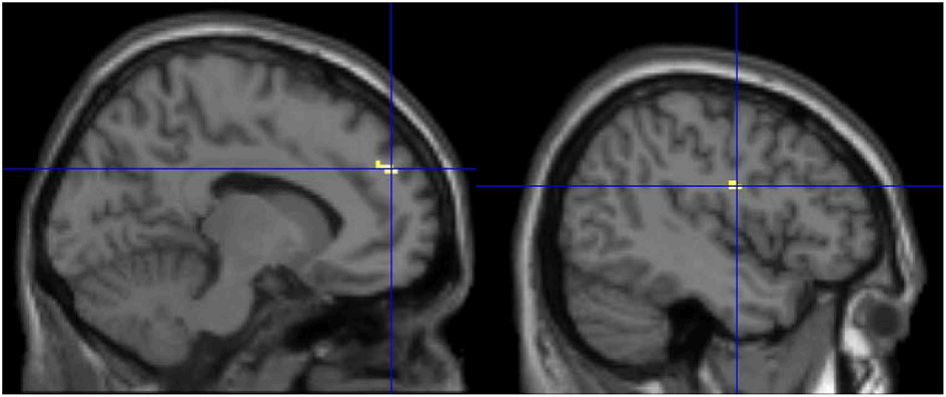
**The increased activation for the block play group compared to the board game group after training**. The increased activation is observed in the medial prefrontal cortex and the precentral gyrus.

The direct comparison of the block play and board game group was also examined for the post-training scan (Figure 4). The results showed that the block play group elicited greater activation in the medial prefrontal cortex and the left precentral gyrus (at a lower extent threshold) than did the board game group after training. There was no difference pre-training difference between groups.

## Discussion

The goal of this preliminary study was to examine the impact of two very different games that have both been suggested to impact spatial reasoning skills—block play and board games. As predicted and as suggested by previous work, both block play and board games were found to result in performance improvements, although in different ways. It should be noted that some improvement may be due to practice effects (performing the same task twice). The same practice effects would be expected to be observed for both groups. While, on average both groups did show faster reaction times and increased accuracy after training, only the block play group showed significant training effects. The neuroimaging results mirrored the behavioral data in that only the block play group showed significant changes in brain activation after training. Together these results provide some support for a differential effect of board games and block play on spatial processing.

Block play has been shown to impact spatial ability in children. In a recent study that examined 847 4- to 7-year-old children it was found that spatial play, including block building and playing with puzzles and board games, was associated with increased spatial ability (Jirout and Newcombe, 2015). Here we found that 5, 30-min structured block play sessions resulted in changes to the neural network responsible for mental rotation as well as increased the speed and accuracy of mental rotation performance. Structured block play in which children build a given structure requires the ability to analyze a spatial representation. It is thought to develop skills in estimation, measurement, patterning, part-whole relations, visualization, symmetry, transformation, and balance (Casey and Bobb, 2003; Stiles and Stern, 2009; Verdine et al., 2014). Blocks Rock! is precisely this type of structured block play game as it requires players to construct a specified structure as accurately and fast as possible. Therefore, it not only encourages accuracy but speed in analyzing and then building the structures.

It was predicted that block play would impact spatial processing; however the specific aspects of spatial processing impacted was not known. The imaging data may provide some hints. Block play training resulted in increased activation in the parahippocampal gyri, cerebellum, and the fusiform gyri. These regions have all been implicated in different aspects of spatial processing (Aguirre et al., 1996; Johnsrude et al., 1999; Aminoff et al., 2007; Stoodley et al., 2010). The parahippocampus has been linked to spatial memory encoding (Johnsrude et al., 1999; Bohbot et al., 2000; Burgess et al., 2001); and spatial navigation (Aguirre et al., 1996; Mellet et al., 2000), particularly the posterior aspect of the region as found here (Aminoff et al., 2007). There cerebellum has also been linked to both spatial and motor processing (Shen et al., 1999; Stoodley et al., 2012) and sensory motor integration (Bastian, 2006; Wiestler et al., 2011), while the fusiform gyrus has been linked to visual object recognition (Chao et al., 1999). All of these processes are involved in structured block play. For example, structured block play is analogous to block copy tasks that have a long history of use in neuropsychology research. Ballard et al. (1992) detailed the intricate hand-eye coordination and spatial memory strategy required to perform a block copy task. In that study it is was found that participants memorized sequences of moves then executed those moves in an iterative pattern moving back and forth between fixating and memorizing and fixating and executing the movement. This requires spatial working memory, sensory-motor processing, and visual object processing. In fact, a similar process is thought to be involved in the solution of a visuospatial problem solving task, the Tower of London (Owen, 2005) with neuroimaging studies of the task showing the involvement of these same brain regions (Dagher et al., 2001; Rowe et al., 2001; Newman et al., 2003).

There are at least two mental rotation strategies that have been identified. One is the holistic strategy in which the image is mentally rotated as a whole, while the other is a piecemeal or viewpoint independent strategy that involves the analysis of the internal relations of image parts (Khooshabeh et al., 2013). Motor simulation is more strongly linked to the holistic strategy. Interestingly, the holistic strategy has been found to be faster and one used by individuals with better visuospatial ability (Cooper, 1975). This suggests that the block play training likely reinforced the holistic strategy resulting in increased motor simulation, as suggested by increased activation of the cerebellum. Further support for this hypothesis comes from the finding that the block play group showed increased activation in the precentral gyrus after training compared to the board game group. The role of the precentral cortex in mental rotation has largely been suggested to be related to motor simulation due to the region's link to motor planning (Cohen and Bookheimer, 1994; Zacks, 2008). In sum, the block play group shows a change in activation in regions linked to both motor and spatial processing while the board game group showed no changes in brain activation. This result raises the possibility that the block play group changed how they were performing the mental rotation task after training. However, it is unclear whether there was a strategy change or whether block play resulted in refining an existing strategy. Further research is required to explore this question.

The board game used here was Scrabble. Previous research has shown that, like block play, board games including word/spelling games may result in improvements in spatial processing (Verdine et al., 2014; Jirout and Newcombe, 2015). This may be particularly true for Scrabble because, as mentioned earlier, spelling is spatial in that the spatial relationships between the letters are important. Additionally, spatial language is used during game play (e.g., up and down). Also, Scrabble may be expected to have an advantage here given that the game involves viewing letters (the same stimuli used in the mental rotation task) in words where the alignment of the letters vary on a different axis on a board, or 2D plane. Lange-Küttner (2009) found that 7-year-old children were sensitive to the spatial axis of the frame in a drawing task; therefore, the practice that Scrabble provides in viewing and rotating words along the x-y axis may be expected to impact spatial processing. However, the board game group here failed to show brain activation changes as a result of training and the improvements in behavioral performance were just under the significance threshold. One possible explanation for the failure to show an effect is the absence of 2D-3D transformation and this absence of letter rotation made the Scrabble game the less powerful training game despite its multiple spatial attributes.

While Scrabble failed to show significant improvements in mental rotation, the hypothesis that board games improve spatial processing cannot be ruled out here. It could be that board game play does benefit spatial processing, but not the processes recruited during mental rotation. Scrabble also has a numerical component with an emphasis on counting. For example, available places must be counted once the game has advanced and many places are occupied. Also free places must be counted to be able to fit the intended word into the empty space. This emphasis on counting and getting objects (words) to fit within a given context may facilitate different spatial processes than block play. Additionally, counting may also recruit verbal working memory resources instead of spatial working memory. Further research that uses a longer training, a larger sample, and/or different spatial tasks are required to determine the precise character of the spatial processing impacted.

## Future directions and limitations

The results from this preliminary study are very promising and suggest that structured block play may be an important tool to help improve spatial processing. However, the results are preliminary in that the number of subjects is rather small. With a larger sample size the behavioral results may become more robust. Additionally, the training period was relatively short. This short training period may have caused the behavioral effects to be small, particularly for the board game group. Also, Blocks Rock! rewards players who complete the structure fastest; therefore the speed of the block building game may have resulted in the children being more attentive. This increase in attentiveness may have made the effects more salient for block play. Further research is necessary with a larger sample and a more extensive training protocol to confirm the results.

The age of the children examined here was chosen based on the literature regarding the development of mental rotation and spatial processing ability (Piaget and Inhelder, 1971; Bialystok, 1989; Kosslyn et al., 1990). It seems that differences in mental rotation appear around 7-years. Also, younger children have difficulty with the types of mental rotation tasks that are typically used in MRI environments. However, it would be important to examine how structured block play may differentially impact mental rotation performance at different developmental stages. Here we found increased activation due to training instead of a decrease in activation that has been previously linked to greater efficiency. We hypothesize that this increase may be related to the age of the participants. In this study the children are still developing mental rotation skills. Therefore, they start with very inefficient strategies that must be improved or even developed. Once the strategy becomes more entrenched and more efficient decreased levels of activation are expected. A longitudinal study in which cognitive ability including spatial ability and spatial working memory is assessed to determine the developmental consequences of block play is necessary to fully characterize the impact block play on spatial ability.

Finally, we failed here to obtain a comprehensive assessment of spatial processing ability in our sample prior to training. Obtaining a measure that explores different tasks and objects with varying complexity including 2D and 3D objects (e.g., the rotated color cube test, Lütke and Lange-Küttner, 2015) is necessary. In addition, using unfamiliar objects may also be important as the use of letters may strongly activate the *what* visual system and interfere with the rotation decision (Lange-Küttner and Küttner, 2015). Some support for this idea comes from a previous mental rotation practice study by Kail and Park (1990). There participants completed 3360 trials of mental rotation on letters and found that rotation experience did not transfer to other objects. It was suggested that the benefits of letter rotation training failed to transfer to other objects because a memory/instance based strategy was used instead of changes to the mental rotation process; therefore, activating the *what* visual system. Some evidence may also be observed here in that post-training activation for the block play group showed increased activation of the fusiform gyrus—part of the *what* processing system—as well as spatial processing regions even though letter stimuli were not included in the block play training. In any case, it may be important in future studies to examine both familiar and unfamiliar objects in the mental rotation task.

## Conclusions

There has been some debate in the literature regarding whether training on one visuospatial task transfers to other tasks. The results presented here suggest that they can. Here training on a speeded, structured block building game, Blocks Rock!, resulted in transfer to mental rotation performance. While it appears that both the block play and board games resulted in some improvements in mental rotation performance the improvements were greater for the block play group. The findings have important implications on child play activities. Given the importance of spatial thinking to success in science, technology, engineering, and mathematics (Newcombe, 2010), using games like structured block building may prove to be important for helping to set a solid foundation.

## Ethics statement

All procedures performed in studies involving human participants were in accordance with the ethical standards of the institutional and/or national research committee and with the 1964 Helsinki declaration and its later amendments or comparable ethical standards.

## Author contributions

SN is the PI and was responsible for most of the writing. MH and AG were responsible for the data collection and analyses and contributed to the writing.

### Conflict of interest statement

The authors declare that the research was conducted in the absence of any commercial or financial relationships that could be construed as a potential conflict of interest. SN has received research grants from the LaCrosse Family Business Trust.
