# Supplementary material for: An fMRI Study of the Impact of Block Building and Board Games on Spatial Ability
Source: Front Psychol. 2016 Aug 29;7:1278. doi: 10.3389/fpsyg.2016.01278 (PMC5002428; doi:10.3389/fpsyg.2016.01278)
Supplement: Supplementary file 1 [file DataSheet1.docx]

**Appendix**

**Parent Survey**

1. Child’s First Name ____________________________________________
2. Child’s DOB _______________ Gender____________________
3. Mother’s highest level of education ______________________________
4. Father’s highest level of education_______________________________
5. Has your child ever been diagnosed with:
6. ADD/ADHD_________ Dyslexia____________ Specific Language Impairment___________
7. Autism____________ Depression__________ Other____________
8. Does your child play a musical instrument No_________ Yes _________
   1. If yes, which instrument_________________
9. How often does/did your child play games with die like Monopoly?
10. How often does/did your child play card games (cards with numbers)?
11. How often does/did your child play block building games (e.g., Legos)?
12. When did you start trying to teach your child to calculate? For example, asking them questions like “If you have 1 and I gave you 2 how many do you have now?”
13. Does your child enjoy math at school?
14. What is your child’s favorite subject in school?
