# Supplementary material for: An fMRI Study of the Impact of Block Building and Board Games on Spatial Ability
Source: Front Psychol. 2016 Aug 29;7:1278. doi: 10.3389/fpsyg.2016.01278 (PMC5002428; doi:10.3389/fpsyg.2016.01278)
Supplement: Supplementary file 2 [file DataSheet2.docx]

**Supplemental Data**

**Table 1: Accuracy 2x2x2 ANOVA SAS Output**

| **Source** | **F-value** | **Pr>F** | **η^2^** |
| --- | --- | --- | --- |
| **group** | 3.36 | 0.069 | 0.025 |
| **diff** | 14.98 | 0.0002 | 0.11 |
| **time** | 5.06 | 0.027 | 0.038 |
| **time*diff** | 0.07 | 0.78 | 0.0006 |
| **group*diff** | 0.76 | 0.39 | 0.0057 |
| **group*time** | 0 | 0.96 | 0 |
| **group*time*diff** | 0.07 | 0.78 | 0.0006 |

Notes: time=pre-training vs. post-training; diff=easy vs. hard; group=block play vs. board game

**Table 2: Reaction Time 2x2x2 ANOVA SAS Output**

| **Source** | **F-value** | **Pr>F** | **η^2^** |
| --- | --- | --- | --- |
| **group** | 1.70 | 0.2 | 0.013 |
| **diff** | 10.48 | 0.0016 | 0.083 |
| **time** | 3.78 | 0.054 | 0.03 |
| **time*diff** | 0.56 | 0.45 | 0.0044 |
| **group*diff** | 0.25 | 0.62 | 0.004 |
| **group*time** | 1.40 | 0.24 | 0.011 |
| **group*time*diff** | 0.77 | 0.38 | 0.0061 |

Notes: time=pre-training vs. post-training; diff=easy vs. hard; group=block play vs. board game

Table 3: Within-subjects ANOVA results

| **Source** | **F Value** | **Pr > F** |
| --- | --- | --- |
| ***Block Play Accuracy*** | | |
| **time** | 5.76 | 0.0309 |
| **diff** | 8.40 | 0.0117 |
| **time*diff** | 1.06 | 0.3203 |
| ***Block Play Reaction Time*** | | |
| **time** | 8.92 | 0.0098 |
| **diff** | 7.25 | 0.0175 |
| **time*diff** | 0.04 | 0.8403 |
| ***Scrabble Accuracy*** | | |
| **time** | 3.51 | 0.0837 |
| **diff** | 17.01 | 0.0012 |
| **time*diff** | 0.00 | 0.9826 |
| ***Scrabble Reaction Time*** | | |
| **time** | 0.48 | 0.4991 |
| **diff** | 14.05 | 0.0024 |
| **time*diff** | 5.75 | 0.0322 |

Notes: time=pre-training vs. post-training; diff=easy vs. hard

Difference analysis: The post-pre training accuracy and RT differences were calculated for each participant. An analysis was performed to determine whether there were group differences. When collapsed across difficulty, a t-test showed differences only for RT [accuracy (p=0.47): block play mean difference= 8.25% ±13%; board game mean difference: 7.85%±16%; RT (p=0.059): block play mean difference: -0.42±0.55; board game mean differences: -0.1±0.54]. Additionally, an analysis was performed that examined each difficulty level separately (see Figure below; error bars are standard error). It appears that the RT effect is driven by the easy condition (p=0.0027) with there being no group difference for the hard condition (p=0.38).
